# Seats for Female Attendants in Stores

**Published:** 1880-09

**Authors:** 


Seats for Female Attendants in Stores.—The subject of Seats for shop women has not received the attention from the Medical Profession that its insportance requires, in this country, or elsewhere. The *Chicago Medical Union*, of a recent date, says: "Following the agitation of this subject, by Mr. Edes in England, and by the *Philadelphia Medical Times*, in the United States, it is said that *ninety* business houses in Dublin have provided the seats." Now let us see if we cannot present an *equal* number in Baltimore by the end of the present year, at least?

